# Long-term Management Effects and Temperature Sensitivity of Soil Organic Carbon in Grassland and Agricultural Soils

**DOI:** 10.1038/s41598-019-48237-7

**Published:** 2019-08-21

**Authors:** Rajan Ghimire, Prakriti Bista, Stephen Machado

**Affiliations:** 1 New Mexico State University, Agricultural Science Center, Clovis, NM USA; 2 Oregon State University, Columbia Basin Agricultural Research Center, Pendleton, OR USA

**Keywords:** Agroecology, Agroecology

## Abstract

Soil organic carbon (SOC) is integral to soil health and agroecosystem resilience. Despite much research, understanding of temperature sensitivity of SOC under long-term agricultural management is very limited. The main objective of this study was to evaluate SOC and nitrogen (N) dynamics under grasslands and winter wheat (*Triticum aestivum* L)-based crop rotations in the inland Pacific Northwest (IPNW), USA, and measure SOC mineralization under ambient and elevated incubation temperatures. Soil samples were collected from 0–10 and 10–20 cm depths from an undisturbed grassland (GP), winter wheat-pea (*Pisum sativum* L) rotations under conventional tillage (WP-CT) and no-tillage (WP-NT), and winter wheat-fallow rotation under conventional tillage (WF-CT) and analyzed for SOC and N pools. Soil samples were incubated at 20 °C and 30 °C for 10 weeks, and SOC mineralization rates were estimated using the first order kinetic model. The GP had the greatest amounts of SOC, total N (TN), and microbial biomass carbon (MBC) and WP rotations had higher inorganic N content than other treatments. The SOC mineralization at elevated incubation temperature was 72–177% more than at the ambient temperature, and the greatest effect was observed in GP. The SOC storage under a given management did not have consistent effects on soil carbon (C) and N mineralization under elevated temperature. However, soil disturbance under WP-CT and WF-CT accelerated SOC mineralization leading to soil C loss. Reducing tillage, integrating legumes into crop rotations, and growing perennial grasses could minimize SOC loss and have the potential to improve soil health and agroecosystem resilience under projected climate warming.

## Introduction

Soils release more than 60 Gt of C to the atmosphere annually as carbon dioxide (CO_2_), seven times more than the amount of CO_2_ released from fossil fuel burning^1^. Climate change is predicted to alter SOC dynamics and thereby affect net agroecosystem C balance^2^. The inland Pacific Northwest (IPNW) of USA is expected to experience a rise in average temperature of 2.9 °C by 2040 and 5.4 °C by 2080 compared to the 1970–1999 average temperature with high variability in temperature extremes during the 2040 and 2080^3^. The predicted warming is expected to alter soil C dynamics and nutrient cycling under natural as well as agricultural systems^4,5^. In an undisturbed system, soil CO_2_ efflux is often balanced by total C input through root production, root exudates, and residue inputs^6^, whereas SOC balance in agricultural soils depend on how crop and soil management practices affect biomass C inputs and soil C loss through organic matter decomposition and erosion^7^. Soil disturbance (tillage) often triggers SOC loss from agricultural soils because it increases soil biological activity and brings organic residues in contact with decomposers^8^. In contrast, reduced- and no-tillage minimize SOC loss. Crop rotation and cropping intensification and diversification increase diversity and abundance of microbial substrates, microbial biomass and activity, and ultimately increase SOC sequestration^9–11^. However, increasing interest in understanding sensitivity of agriculture sector to increased climate change and variability and improving soil health and resilience underscores the need for more research on how long-term management and potential changes in soil temperature affect SOC dynamics under diverse management systems.

Long-term experiments provide valuable information required for understanding management effects on SOC dynamics and such information is a prerequisite for designing sustainable agroecosystems^12,13^. The Pendleton long-term experiments (PLTEs), some of which date back to 1931, have provided a wealth of knowledge on how long-term tillage, crop residue, and cropping systems management influenced SOC and N dynamics and dryland wheat production in the IPNW^12,14,15^. The PLTEs compare diverse crop rotations, tillage, crop residues, and nutrient management practices^15^. The soils under the diverse management practices may respond differently to projected climate change, and the PLTEs could be a valuable resource to understand how alternative management practices feedback to changes in climatic variables (i.e., temperature and precipitation) to influence the agricultural sustainability.

Soil warming could accelerate soil respiration and SOC loss because the rise in temperature enhances soil microbial activity and accelerates residue decomposition^5,16–18^. However, response of SOC to soil warming vary with agroecosystem type, diversity in root and residue inputs, and disturbance level^6,19^. Typically, grassland systems sequester more SOC than agricultural soils, but the SOC is sensitive to disturbance that accrued SOC is easily lost when the grasslands are cultivated^12,20^. The decline in SOC will continue, particularly under intensively tilled crop-fallow systems, until a new equilibrium is reached^21^. In contrast, alternative management systems that contribute to belowground C and nutrient inputs and reduce soil disturbance can accumulate SOC^7,22^. Studies show that reduced tillage and higher cropping intensity lowers soil temperature^23^ and favors SOC sequestration. There is inconsistency in the literature about how SOC accumulated under alternative management systems responds to seasonal climate variability and projected climate change^19,24^. Currently, at least a 10 °C increase in summer temperatures compared to winter temperatures is observed in the IPNW of USA^25^. Seasonal temperature differences are expected to increase with climate change, which will undoubtedly influence soil microbial activity and SOC storage^7,17^. Besides, precipitation and evapotranspiration are expected to change by the end of the century in the IPNW^26^, which will affect crop yield and biomass C input^27^. Cultivated soils in the dryland IPNW have already lost 30–60% of SOC in the past century. Soil warming could accelerate SOC loss and subsequently affect agricultural productivity^12,20^.

It is difficult to assess the extent to which projected changes in temperature and precipitation affect the regional agriculture because the effects of climate change are confounded with seasonal and inter-annual variability of weather parameters^28^. Designing controlled experiments to evaluate the role of individual factors (e.g., temperature) on SOC dynamics may provide information on the relative response of diverse management systems to changes in climatic variables^19^. Integrating mineralization kinetic models in controlled laboratory studies could further explain the decomposition characteristics of SOC. The first-order kinetic model has been used for estimating the decomposition characteristics of organic materials, specifically *C*_0_, the labile fraction of SOC that is sensitive to changes in management or the environment, and *k*, the decomposition rate constant^14^. This information is necessary to facilitate the management changes needed to improve agricultural sustainability under changing climate. Specifically, information on the effect of elevated temperature on SOC dynamics in dryland agroecosystems in the IPNW is lacking.

The main objectives of this study were to i) evaluate chemical and biological soil properties at 0–10 cm and 10–20 cm depths under selected long-term plots of the PLTEs representing diverse crop rotations and tillage systems, ii) evaluate SOC mineralization at 20 °C and 30 °C temperatures under these treatments, and iii) model the temperature sensitivity of SOC mineralization using a first-order kinetic model.

## Results

### Soil chemical and biological properties

Soil pH was significantly different among treatments, while SOC, TN, and inorganic N contents varied with treatment × soil depth interaction (Table S2). Soil pH averaged for two depths was significantly greater under GP (6.4) than under all wheat-based rotations (WP-NT, WP-CT, and WF-CT) in which soil pH ranged from 4.8 to 5.2. The SOC content was 78% greater in 0–10 cm than in 10–20 cm depth in GP, but it was not significantly different between soil depths in WP and WF treatments (Table 1). In 0–10 cm depth, SOC content was highest under GP, which was not significantly different from WP-NT but was significantly greater than WP-CT and WF-CT. In 10–20 cm depth, SOC content ranged between 10.8 to 14.3 g kg^−1^ and was not significantly different among treatments.

**Table 1:** Chemical and biological properties of soils under different treatments for the laboratory incubation study.

Soil depth	Treatment^†^	Soil pH	SOC (g kg^−1^)^‡^	TN (g kg^−1^)	Inorganic N (mg kg^−1^)	MBC mg kg^−1^
0–10 cm	GP	6.2 ± 0.04	24.6 ± 1.73 aA	1.90 ± 0.12 aA	22.6 ± 0.69bA	296 ± 4.44 aA
	WP-NT	4.9 ± 0.19	18.2 ± 1.40abA	1.35 ± 0.07abA	37.4 ± 2.43 aA	142 ± 10.1bA
	WP-CT	5.1 ± 0.14	14.1 ± 0.05bcA	0.94 ± 0.02bcA	15.1 ± 0.97cA	138 ± 12.1bA
	WF-CT	4.8 ± 0.04	10.8 ± 0.33cA	0.70 ± 0.01cA	5.63 ± 1.01dA	36.0 ± 2.52cA
10–20 cm	GP	6.5 + 0.06	13.8 ± 0.29aB	1.07 ± 0.06aB	1.24 ± 0.06cB	204 ± 11.2aB
	WP-NT	5.2 ± 0.27	13.9 ± 2.70 aA	0.94 ± 0.19 aA	14.3 ± 0.21aB	118 ± 9.25bA
	WP-CT	5.1 ± 0.09	14.3 ± 0.68 aA	0.98 ± 0.07 aA	15.1 ± 1.38 aA	128 ± 11.3bA
	WF-CT	4.9 ± 0.05	10.8 ± 0.39bA	0.71 ± 0.03 aA	7.59 ± 0.91bA	56.1 ± 8.20cA

^†^GP, grass pasture; WP-NT, wheat-pea rotation under no-tillage; WP-CT, wheat-pea rotation under conventional tillage; and WF-CT, wheat-fallow rotation under conventional tillage. SOC, soil organic carbon; TN, total nitrogen; and SMBC, soil microbial biomass carbon.

^‡^Same lowercase letters in a column (mean ± standard error) indicate no significant difference between treatments within a depth and same uppercase letters in a column indicate no significant difference between soil depths (p = 0.05, Tukey test).

Soil TN content in 0–10 cm depth was highest in GP (1.9 g kg^−1^), which was not significantly different than WP-NT but was significantly higher than WP-CT and WF-CT. Soil TN content in 10–20 cm depth ranged between 0.71 to 1.07 g kg^−1^ and was not significantly different among treatments. Soil inorganic N content was about 18 and 2.6 times greater in 0–10 cm depth than in the 10–20 cm depth in GP and WP-NT, respectively, and it was not significantly different between soil depths in WP-CT and WF-CT (Table 1). Soil inorganic N content in 0–10 cm depth was significantly greater in WP-NT than other treatments. Soil inorganic N content in 10–20 cm depth, on the other hand, was significantly greater under WP-CT and WP-NT than under GP and WF-CT. Soil inorganic N in 10–20 cm depth was only 1.24 mg kg^−1^ in GP, significantly lower than soil inorganic N content in the same soil depth in all wheat-based rotations (7.59–14.3 mg kg^−1^).

There was a significant treatment × depth interaction on soil MBC (Table S2). Soil MBC content was significantly greater in 0–10 cm depth (296 mg kg^−1^) than in 10–20 cm depth under GP (204 mg kg^−1^), whereas it was not significantly different between the soil depths in wheat-based rotations (Table 1). Among wheat-based rotations, MBC content was significantly greater in WP rotations (WP-NT and WP-CT) than in WF-CT.

### Soil carbon and nitrogen mineralization

**Figure 1:**
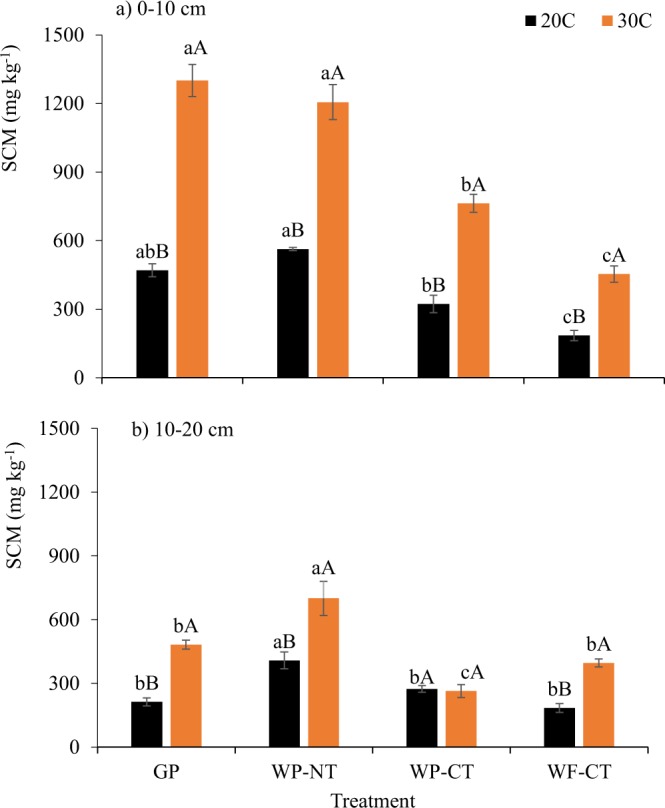
Soil carbon mineralization (SCM) at 20 °C and 30 °C temperatures. GP, grass pasture; WP-NT, wheat-pea rotation under no-tillage; WP-CT, wheat-pea rotation under conventional tillage; and WF-CT, wheat-fallow rotation under conventional tillage. Error bars indicate standard error, same lowercase letters indicate no significant difference between treatments within an incubation temperature and same upper case letters indicate no significant difference between incubation temperatures within a treatment (p = 0.05, Tukey test).

Significant treatment × depth interactions on soil C mineralization (SCM) were observed during the 70-days incubation period (Table S3). In 0–10 cm depth, SCM under WP-NT was comparable with GP, but it was significantly greater than under WP-CT and WF-CT under both temperatures (Fig. 1). Soil incubation under elevated temperature increased SCM by 125 to 177% compared to SCM under ambient temperature. The greatest increase in SCM at higher incubation temperature was observed in GP, followed by WF-CT, WP-CT, and WP-NT. The WP-NT had significantly greater SCM than all other treatments in 10–20 cm depth, and the effects of elevated temperature were not consistent across treatments. In this depth, elevated temperature resulted in higher cumulative SCM in GP, WP-NT, and WF-CT by 72 to 126% compared to ambient condition, while no effects were observed in WP-CT.

**Figure 2:**
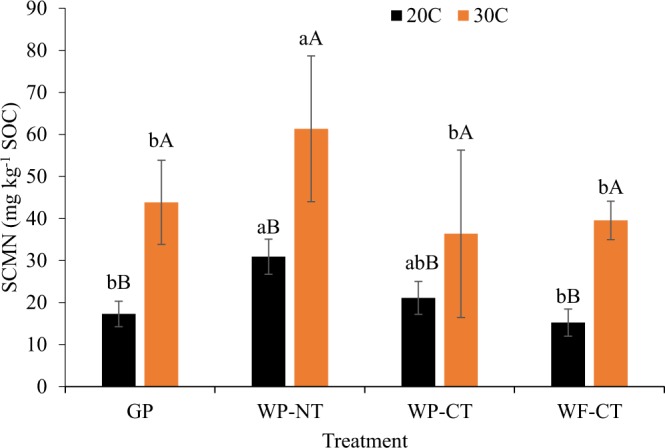
Soil temperature effects on soil carbon mineralization normalized to soil organic carbon in 0–20 cm depth. GP, grass pasture; WP-NT, wheat-pea rotation under no-tillage; WP-CT, wheat-pea rotation under conventional tillage; and WF-CT, wheat-fallow rotation under conventional tillage. SCMN, soil carbon mineralization normalized to soil organic carbon. Error bars indicate standard error, same lowercase letters indicate no significant difference between treatments within an incubation temperature and same upper case letters indicate no significant difference between incubation temperatures within a treatment (p = 0.05, Tukey test).

The SCM normalized to SOC (SCMN) was significantly influenced by cropping systems treatment, temperature, depth, and treatment × temperature interaction (Table S3). Overall, SCMN was 36% greater in 0–10 cm depth than in 10–20 cm depth, and it was 114% greater under elevated temperature (30 °C) than under ambient temperature (20 °C) (Fig. 2). The SCMN was not significantly different between WP-NT and WP-CT under ambient temperature, but SCMN under these treatments was significantly greater than SCMN under GP and WF-CT. Under higher incubation temperature, SCMN was significantly greater under WP-NT than other treatments. The temperature response, however, was highest in WF-CT with 159% greater SCMN at 30 °C than at 20 °C followed by 153% in GP, 99% in WP-NT, and 72% in WP-CT.

**Figure 3:**
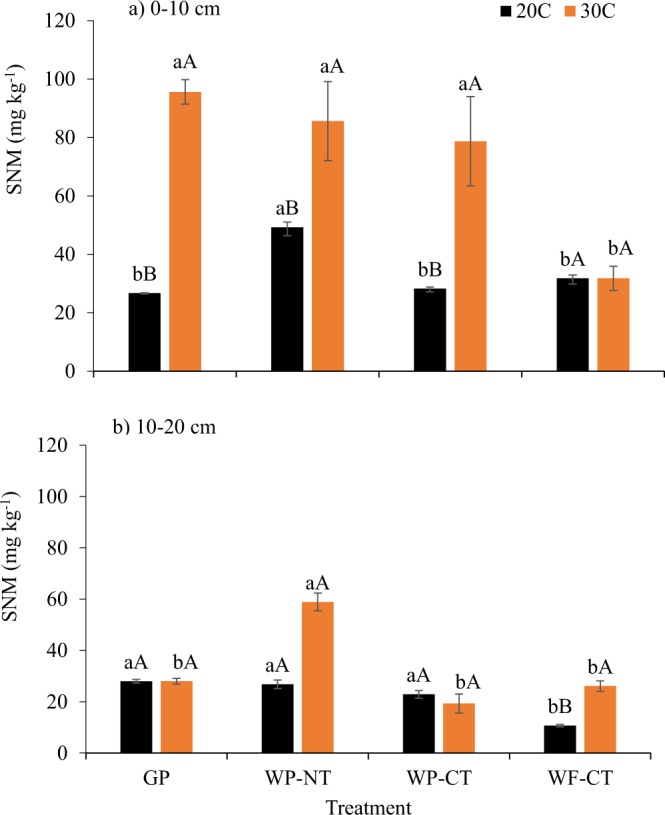
Soil nitrogen mineralization (SNM) at 20 °C and 30 °C temperatures. GP, grass pasture; WP-NT, wheat-pea rotation under no-tillage; WP-CT, wheat-pea rotation under conventional tillage; and WF-CT, wheat-fallow under conventional tillage. Error bars indicate standard error, same lowercase letters indicate no significant difference between treatments within an incubation temperature and same upper case letters indicate no significant difference between incubation temperatures within a treatment (p = 0.05, Tukey test).

Significant treatment × temperature × depth interactions on soil nitrogen mineralization (SNM) were observed (Table S3). In 0–10 cm depth at ambient temperature, SNM was significantly greater under WP-NT (49.3 mg kg^−1^) than all other treatments (Fig. 3). The SNM among the other treatments was not significantly different and was in the range of 26.7 to 31.8 mg kg^−1^. With elevated temperature, SNM increased significantly in GP, WP-NT, and WP-CT. There was no significant difference in SNM between the ambient and elevated temperature in WF-CT. The SNM under elevated temperature was highest under GP, followed by WP-NT, WP-CT; SNM under these treatments was significantly greater than SNM under WF-CT. At ambient temperature, SNM in the 10–20 cm depth was considerably lower in WF-CT than in all other treatments, and SNM content did not differ among GP, WP-NT, and WP-CT at ambient temperature. At elevated temperature, SNM under WP-NT was significantly higher than SNM under other treatments in the 10–20 cm soil depth profile. At this temperature, no significant differences in SNM were observed among the GP, WP-CT, and WF-CT treatments (Fig. 3).

**Figure 4:**
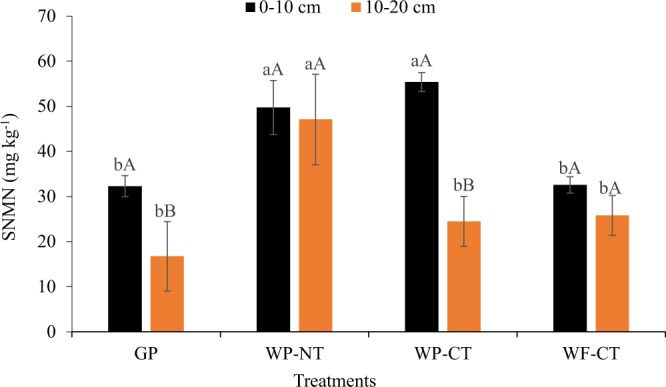
Soil nitrogen mineralization normalized to total nitrogen (SNMN) at 0–10 cm and 10–20 cm depths under different management systems. GP, grass pasture; WP-NT, wheat-pea rotation under no-tillage; WP-CT, wheat-pea rotation under conventional tillage; and WF-CT, wheat-fallow rotation under conventional tillage. Error bars indicate standard error, same lowercase letters indicate no significant difference between treatments within a soil depth and same uppercase letters indicate no significant difference between soil depths within a treatment (p=0.05, Tukey test).

There were significant temperature and treatment × depth interaction effects on soil N mineralization normalized to total N (SNMN) (Table S3). The SNMN content was significantly greater at elevated temperature than at ambient temperature under all treatments. The SNMN content was significantly greater under WP-CT and WP-NT than GP and WF-CT in 0–10 cm depth whereas it was significantly greater in WP-NT than all other treatments in 10–20 cm depth (Fig. 4). Compared between soil depths, SNMN was significantly greater in 0–10 cm than 10–20 cm depth in GP and WP-CT, and it was not different between soil depths in other treatments.

### Soil carbon mineralization kinetics

**Figure 5:**
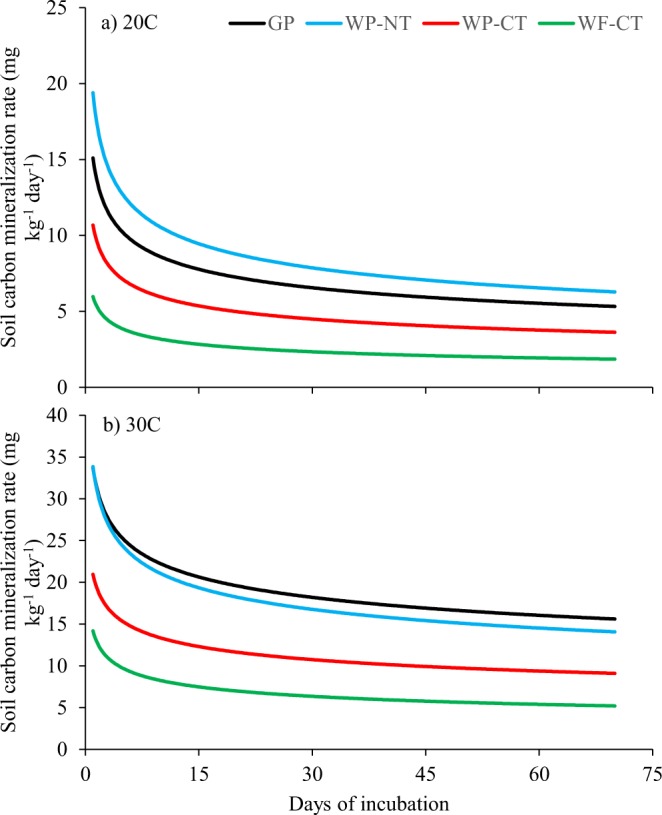
Soil carbon mineralization rate at 20 °C and 30 °C under different treatments at 0–10 cm soil depth using a first-order kinetic model. GP, grass pasture; WP-NT, wheat – pea rotation under no-tillage; WP-CT, wheat-pea rotation under conventional tillage; and WF-CT, wheat-fallow rotation under conventional tillage.

High incubation temperature stimulated SOC mineralization across all treatments during the 70-day incubation period. The first-order kinetic model predicted SOC mineralization with a good fit as indicated by high *r* and low root mean square error (RMSE) and normalized root mean square error (NRMSE), specifically at 0–10 cm depth (Fig. 5, Table 2). The SOC mineralization rate was considerably higher in the first week followed by a gradual decrease for the next few weeks and ultimately leveling off across all treatments after four weeks of incubation. Comparing *C*_0_, the labile SOC fraction, and *k*, the decomposition rate constant, between treatments revealed that *C*_0_ was dependent on crop residue inputs as well as soil temperature, and *k* was dependent on soil temperature. The *C*_0_ was 153%, 225%, and 79% greater in GP, WP-NT, and WP-CT, respectively than in WF-CT at ambient temperature, and it was 135%, greater in GP and WP-NT and 47% greater in WP-CT at the elevated temperature. However, *k* was 27% lower at elevated temperature than at ambient temperature and did not differ significantly between treatments. The SOC mineralization increased under elevated temperature, but model parameters were not significantly different between treatments at 10–20 cm depth (data not presented).

**Table 2:** Model parameters (*C*_*o*_ and *k*), correlation coefficient (*r*), root mean square error (RMSE), and normalized root mean square error (NRMSE) of the first-order kinetic model fitted for different treatments at 0–10 cm depth.

Temperature (°C)	Treatment	*C*_*o*_	*K*	*r*	RMSE	NRMSE
20	GP	15.1	0.245	0.95	0.96	11.6
	WP-NT	19.4	0.265	0.95	1.32	12.9
	WP-CT	10.7	0.254	0.93	0.91	15.6
	WF-CT	5.97	0.275	0.80	1.01	31.5
30	GP	33.7	0.181	0.88	3.22	14.9
	WP-NT	33.8	0.207	0.86	3.33	16.3
	WP-CT	20.9	0.196	0.98	0.74	5.80
	WF-CT	14.2	0.236	0.94	0.94	11.9

^†^GP, grass pasture; WP-NT, wheat-pea rotation under no-tillage; WP-CT, wheat-pea rotation under conventional tillage; and WF-CT, wheat-fallow rotation under conventional tillage. SOC, soil organic carbon; TN, total nitrogen; and SMBC, soil microbial biomass carbon.

## Discussion

This study revealed the benefit of reducing soil disturbance and integrating legumes under current condition and projected warming. Soil warming can increase soil microbial activity and thereby accelerate organic residue decomposition. We observed higher SOC mineralization under elevated incubation temperature than ambient temperature across the agricultural systems and grassland. However, the relative SOC mineralization rate in elevated temperature compared to ambient temperature was lower in WP rotations (WP-NT and WP-CT) than grassland or WF-CT. Increase in the fresh organic matter and quality due to improved management (e.g., no-tillage, legume integration) can affect microbial utilization of substrates^29^ and resulting changes in SOC storage. Some microorganisms are better adapted to climate change and variability than others, which affect their substrate utilization and thereby SOC dynamics in grasslands and agricultural soils^17^. In-field response of soil warming also varies with soil type, environmental conditions, and crop management practices^24^. Nevertheless, the relative difference in SOC mineralization under high incubation temperature suggests that diversified systems lose less C than grass-only systems under projected warming conditions.

Long-term SOC balance in agroecosystems depends on how global warming alter C inputs, outputs, and residence time in the soils. High temperature can increase plant biomass production in the temperate ecosystems, leading to greater C inputs to the soil and potentially higher microbial biomass and activity^30^. Studies from IPNW reveal high variability in precipitation and temperature with potential for an overall increase in precipitation and decrease in evapotranspiration in most of the wheat producing area^26^, and total grain yield by the 2070 s in the region is projected to increase in the range of 18–48% (RCP 4.5) and 30–65% (RCP 8.5) compared to the baseline scenario^27^. Under such conditions, C input and microbial activity are expected to increase, which can accelerate SOC mineralization and lead to SOC loss via CO_2_ emissions to the atmosphere if appropriate conservation practices are not adopted^7^. The greater SOC and N stocks we observed under GP and WP-NT than under WP-CT and WF-CT suggested that agricultural systems that minimize soil disturbance, maintain permanent ground cover, and include legumes in the rotation have the potential to restore SOC lost due to intensive cultivation in drylands of IPNW.

While we observed the increase in labile SOC fractions with elevated incubation temperature, the decomposition rate constant (*k*) was decreased, suggesting that SOC inputs and management practices play a critical role in stabilizing SOC under warming conditions. More importantly, the difference in SOC mineralization coefficient (*C*_0_) was smaller under WP rotations than under GP and WF-CT, which also suggests the greater potential of stabilization of labile C accrued under legume-integrated systems compared to grass-only systems. The difference between response of legume-integrated and grass-only systems was also observed in SCMN, an indicator of microbial substrate quality. Diverse crops produce a variety of microbial substrates^10,31^, and each of these compounds has their own kinetic properties. The response of these compounds may vary under soil warming depending on the gross kinetic energy for the substrate decomposition and shifts in the microbial community structure^24^. Crop residues not protected in soil aggregates or freshly released C compounds are more sensitive to elevated temperature than SOC that is stabilized or protected in the soil aggregates^6^. Greater C inputs through root and aboveground residues and associated microbial community, along with reduced- or no soil disturbance under GP and WP-NT, could produce a greater amount of labile substrates and ultimately increase SOC sequestration.

Information is limited on the effects of elevated temperature on N dynamics compared to SOC dynamics. It appears soil N responds differently to SOC under warming conditions than under ambient conditions. There was no response of SNM and SNMN to an increase in incubation temperature, possibly due to a different rates of microbial C and N utilization or greater use of N due to increased microbial biomass and activity under elevated temperature^17^. The significant differences in SNM under higher incubation temperature, mainly in 0–10 cm depth of GP and WP rotations and 10–20 cm depth of WP-NT and WF-CT suggested that soil N from the root and aboveground biomass decomposition is more likely to mineralize due to soil warming. It appears that SOC dynamics under projected warming in IPNW will be N-limited and the relative response will vary with cropping system. More research will reveal the effects of diverse management practices on soil N dynamics under warming conditions. Similarly, further research on soil pH, soil microbial community structure and their influence on nutrient cycling under range of temperatures would help IPNW farmers to understand potential changes in agroecosystems and their impacts to crop production. This study, conducted at the oldest long-term experiment in the western US, provides valuable information on potential changes in soil C and N dynamics under project climate change and variability in the region.

Long-term observations at PLTEs reveal loss of SOC stock by 50% in WF-CT systems^12,20^ while maintaining WP-CT and WP-NT for more than 51 years restored 13% and 30% SOC and 20% and 42% TN, respectively, under these systems. Ogle *et al*.^32^ reported up to 21% recovery of SOC by adopting conservation tillage systems in fields that were previously tilled. The SOC content was the highest in GP, the grassland system that has not been disturbed for more than 80 years, and the lowest in WF-CT, an intensively tilled winter wheat-fallow system. Inorganic N, on the other hand, was greater in WP-NT than all other treatments, potentially because no-tillage provided a more favorable environment for N cycling than conventional system. No-tillage maintains a consistent soil environment for microbial growth and activity, conserves soil moisture, and helps in the soil aggregation that ultimately leads to improved nutrient storage and cycling^32–34^. Pea in rotation may have accumulated inorganic N in the soil through atmospheric N fixation, and no-tillage management conserved the accrued N. Studies show that legumes in rotation support crop production through atmospheric N fixation^35^. Since we did not have a non-legume fallow replacement crop, it’s hard to distinguish whether N accumulation in WP-NT was due to N fixation by legume or due to fallow replacement. Van Kessel and Hartley^36^ reported that including pea in crop rotation or intercropping with other crops could replace up to 84% of the N needed for the following crop. Our observation of two to three times more inorganic N in 0–20 cm soil profile supports the previous observations of improved N availability and increased crop production in legume-based crop rotations.

Adoption of no-tillage and more intensive crop rotations has potential to improve soil health and resilience through their positive effects on microbial substrate availability and quality, microbial growth, SOC accumulation, and nutrient cycling^10,34,37,38^. Average and maximum summer temperatures in IPNW were used to evaluate the maximum potential changes on SOC mineralization, which may not exactly reflect the projected climate change scenarios, but this study provides insights on SOC and N responses to the potential impact of warming and seasonal temperature changes in the labile SOC content. In fact, there is a discrepancy in the literature about how and the extent to which soils and agroecosystems will respond to projected climate change. The observed increase in labile SOC with a decrease in decomposition rate constant (*k*) under elevated temperatures compared to ambient temperature emphasizes the need for conservation systems that reduce soil disturbance and maintain vegetation or surface residue cover. Such systems have the potential to sequester SOC by the physical protection of organic matter from increased microbial activity, chemical protection by stabilization of root and shoot residue-derived organic compounds in the soil, support of more fungal than bacterial growth, and biochemical protection of root exudates^24,39^. Soils from diverse management systems were exposed to the same experimental condition (temperature gradient, moisture), which allowed us to compare relative response of diverse management practices to potential warming. Increasing crop diversity by including pea in crop rotation appears to reduce SOC loss under projected climate change and has the potential to mitigate climate change impacts in dryland agriculture in IPNW.

## Conclusion

This study examined soil properties at surface and subsurface soils under diverse crop rotation and tillage management systems and estimated the temperature sensitivity of SOC mineralization in surface soil using a first-order kinetic model. Increased SOC loss under elevated temperature compared to ambient temperature suggested high sensitivity of SOC to projected climate warming. Soil C mineralization increased with increasing incubation temperature. However, potential effects of warming varied with tillage management and cropping systems. Traditional tilled crop-fallow system substantially depleted SOC that also appears to be more vulnerable to warming. A smaller difference in C mineralization rate in WP-NT than other treatments, specifically the difference in *C*_0_, between ambient and elevated incubation temperatures indicated the potential for SOC sequestration in no-tilled legume-integrated systems under projected warming conditions. No-tillage and diversified crop rotations that integrate legume have a potential to improve soil health and reduce negative feedback to projected climate change in semiarid agroecosystems such as IPNW of USA.

## Materials and Methods

### Study site and treatments

The study was conducted at the Columbia Basin Agricultural Research Center (CBARC) near Pendleton, OR, USA (45°42′N, 118°36′W, and 438 m elevation). The study area has a Mediterranean-type climate with cool wet winters and hot, dry summers. The 83-year average (1931–2013) annual precipitation was 421 mm, and the maximum and minimum temperatures were 17.6 and 3.04 °C, respectively (Table S4). About 70% of the total annual precipitation occurs between September and March^12^. All the experimental plots were within 500-m distance from each other and had similar soil characteristics; soils are classified as a Walla Walla silt loam (coarse-silty, mixed, superactive, mesic Typic Haploxerolls), which is generally a well-drained soil consisting of loess deposits overlying basalt^40^.

Soil samples for this study were collected from selected long-term plots of the PLTEs. The treatments selected represented management practices differing in soil disturbance, cropping intensity, and SOC concentration (Table S1). The perennial grass pasture (GP) field was established in 1931 and has not been disturbed since then. The GP plot was a 46 × 109 m field with native grasses such as blue-bunch wheatgrass (*Agropyron spicatum* Pursh) and Idaho fescue (*Festuca idahoensis* Elmer) as dominant species. The GP received occasional controlled light grazing until 1985 and has not been grazed since then, but no external fertilizer or biomass inputs have been applied since 1931. Vegetation was sometimes clipped after summer to allow plant regrowth in the following season. The winter wheat-pea rotations (WP-CT and WP-NT) were established in 1964 as a part of a randomized complete block experiment with four replications. Both phases of crop rotations were present each year, allowing for collection of agronomic and environmental data for each crop every year. Tillage management has been modified over time as new tillage equipment became available^41^. The WP-CT plots were tilled five to six times within a two-year crop cycle. These plots were moldboard plowed in the fall to a depth of 15 to 18 cm followed by secondary tillage to a depth of approximately 10 cm. Pea plots were cultivated one to three times annually to a depth of approximately 10 cm with a spring-tooth cultivator (John Deere CC, Moline, IL) and the plots were roller-packed using a Dunham Culti-packer (Dunham Co., Dunham, OH) after planting pea. The WP-NT plots were mowed after harvest and surface tilled to a depth of 3–4 cm using a Dunham skew-treader (Dunham Co., Dunham, OH) to breakdown wheat residues to facilitate pea sowing. No mowing or skew-treading was done before sowing wheat into the pea stubble. Herbicides were applied as needed to control weeds in WP-CT and WP-NT plots. The conventionally tilled winter wheat-summer fallow (WF-CT) treatment was established in 1940 and has used moldboard plow tillage since then^15,42^. These plots were part of another randomized complete block experiment with three replications. Primary tillage in the WF-CT was implemented in the spring (late March to early April) of the fallow year. Soils were tilled to a depth of 20 cm, leaving about 7% residue cover. The WF-CT plots were cultivated to a depth of 10 to 15 cm using a field cultivator and harrow. These plots receive five to six tillage passes in a crop rotation cycle.

### Soil sampling and laboratory analysis

Soil cores were collected from 0–10 cm and 10–20 cm depths of the WP-CT, WP-NT, and WF-CT cropping systems and from three randomly selected three locations within the GP field using a core sampler (inner diameter 1.85 cm) in summer of 2015. At least four soil cores were collected from each plot, composited by depth increment, thoroughly homogenized, and brought to the laboratory for long-term incubation as well as for soil microbial biomass, SOC, N, and soil pH analyses.

In the laboratory, soil samples were stored in a refrigerator (4 °C) for soil microbial biomass and potential soil C mineralization (SCM) measurements. All visible plant materials (roots, stems, and leaves) and crop residues were removed, and the soil was sieved using a 2-mm sieve before laboratory analysis. Soil water content was determined gravimetrically by oven drying 10-g soil samples at 105 °C for 24 hr and water content was calculated as a difference between the wet and dry weight of soil samples. Soil pH was measured using an Orion Star A215 pH/conductivity meter (Thermo Fisher Scientific Inc., Beverly, MA, USA) on 1:2 soil to 0.01 M CaCl_2_ extracts after a 30-min equilibration time^43^. Total C and N contents were determined by dry combustion analysis (Flash EA 1112 series, Thermo Finnigan, San Jose, CA) of oven-dried (60 °C, 72 hr) and finely ground (<0.05 mm) soil samples. Soils were ground in a Shatter 1 Box 8530 ball mill (Spex Sample Prep., Metuchen, NJ) for 2 min. Soil pH, which was less than 6.6 in all samples, was used to indirectly detect inorganic C. Studies show that total C in soils with pH < 7.4 is mostly SOC^44^. Therefore, total C content in soil was considered to be SOC. Soil inorganic N was analyzed as the sum of KCl- extractable NO_3_^−^N and NH_4_-N in an autoanalyzer (Astoria-Pacific, Inc. Clackamas, OR). For this, 5 g of soil were extracted with 25 ml of 1 M KCl solution. Soil microbial biomass C (MBC) was determined by the fumigation incubation method^45^ in which approximately a 10-g soil (dry weight equivalent) sample was fumigated in a vacuum desiccator for 48 hr using chloroform, and the samples were incubated for a week in 1-L glass jars. The headspace CO_2_-C released from samples during a week-long incubation was determined by a single-cell IRGA system (specifically, the LI-820 CO_2_ analyzer by LI-COR Biosciences, Lincoln, NE)^46^. An efficiency factor of 0.41 was used for MBC calculations without subtracting to non-fumigated control^33^. Long-term soil C mineralization was analyzed by aerobic incubation of approximately 20 g of soil in 1-L glass jars for 70 days, and CO_2_-C released from the incubated samples were measured in LI-820. For this, soil samples were weighed in plastic specimen cups, brought to field capacity (23% g/g water content), and incubated at 20 °C and 30 °C in an HC30 accelerated aging chamber incubator (Hoffman Manufacturing, Inc. Corvallis, OR) in a dark place, and CO_2_-C released from the soil samples was measured with LI-820 after 24 h, 3 days, 8 days, and 14 days, and on a weekly interval thereafter. After each gas sampling, CO_2_ concentration and air pressure were re-equilibrated by opening the lids of incubation jars and flushing with a vacuum air pump (Ted Pella Inc. Redding, CA). The specimen cups were weighed every week to determine the soil moisture loss, and the soil water content was brought to 23% by adding deionized water. The SNM was determined as inorganic N on soil samples incubated for SCM. The selected incubation temperature for SCM and SNM represents the maximum possible increases in soil temperature in the IPNW region of USA^3^. These temperatures are close to long-term annual average soil surface air temperature (19.6 °C) and a maximum summer soil surface air temperature (29.3 °C, i.e., long-term average of June-August) at the experimental site.

### Statistical analysis and modeling

Data for soil properties were tested for normality and homogeneity of variance and analyzed statistically by using a MIXED procedure of SAS for randomized complete block experiments with a split-plot arrangement (v.9.3, SAS Institute, Cary, NC). Soil pH, SOC, TN, inorganic N, and MBC data were analyzed considering treatments as the main plot (fixed) factor, soil depths as a split-plot (fixed) factor, and replication as a random factor. Cumulative SCM and SNM data were analyzed using treatments, incubation temperature, and soil depths as fixed factors and replication as a random factor. Since SOC and TN contents in soil were not consistent in all treatments, the cumulative SCM and SNM data were normalized to SOC and TN content, respectively, and analyzed using the same procedure. Treatment means differing in F-test were separated using the LSMEAN procedure of SAS. Statistical significance was evaluated at *P* ≤ 0.05 unless otherwise stated. Additionally, the observed SOC mineralization rates (*C*_*min*_) after 70 days of incubation were fitted to the first-order kinetic model that describes SOC mineralization as a function of labile SOC content and decomposition time.

1 $${C}_{min}={C}_{0}.{t}^{-k}$$

where, *C*_*min*_ is SOC mineralization rate, *C*_0_ is the labile pool SOC (mg C kg^−1^), *k* is the decomposition rate constant (*day*^*−1*^), and *t* is the decomposition time in days. The model parameters were estimated by pooling the observed SOC mineralization data over three replications. Model performance under each treatment variable was compared using Pearson’s correlation coefficient (*r*) between the observed and predicted values, RMSE, and NRMSE. The RMSE was estimated as a mean squared error between the model-predicted values and the observed values, and NRMSE as a RMSE value standardized with the means of the observed values^21^. A good model fit has a greater *r* value and lower RMSE and NRMSE.

## Supplementary information

Table S1-S3

Table S4

## Data Availability

All the data used in this paper are displayed in tables and figures. Treatment details and analysis of variance information are provided as a supplementary document.
