# Supplementary material for: Long-term Management Effects and Temperature Sensitivity of Soil Organic Carbon in Grassland and Agricultural Soils
Source: Sci Rep. 2019 Aug 21;9:12151. doi: 10.1038/s41598-019-48237-7 (PMC6704134; doi:10.1038/s41598-019-48237-7)
Supplement: Supplementary file 1 — Table S1-S3 [file 41598_2019_48237_MOESM1_ESM.docx]

**Supplementary tables: Long-term Management Effects and Temperature Sensitivity of Soil Organic Carbon in Grassland and Agricultural Soils**

Rajan Ghimire^1*^, Prakriti Bista^2^, and Stephen Machado^2^

^1^New Mexico State University, Agricultural Science Center, Clovis, NM.

^2^Oregon State University, Columbia Basin Agricultural Research Center, Pendleton, OR.

*Correspondence: [rghimire@nmsu.edu](mailto:rghimire@nmsu.edu), +1, 575-985-2292.

**Table S1**. Treatments history of the experimental plots from where the soil samples were collected for the laboratory incubation study.

| Treatment | Crops | Tillage | Period under the current management (Year) |
| --- | --- | --- | --- |
| GP | Perennial grasses | No-tillage | 84 |
| WP-NT | Winter wheat-pea rotation | No-tillage | 51 |
| WP-CT | Winter wheat-pea rotation | Conventional tillage | 51 |
| WF-CT | Winter wheat-summer fallow | Conventional tillage | 74 |

**Table S2**. Analysis of variance (p-values) for chemical and biological properties of soils used in the laboratory incubation study.

| Parameter† | Treatment (T) | Depth (D) | T x D |
| --- | --- | --- | --- |
| Soil pH | <0.001 | 0.37 | 0.76 |
| SOC | 0.002 | 0.01 | 0.04 |
| TN | 0.002 | 0.009 | 0.005 |
| Inorganic N | 0.001 | <0.001 | <0.001 |
| SMBC | <0.001 | 0.16 | 0.004 |

†SOC, soil organic carbon; TN, total nitrogen; SMBC, soil microbial biomass carbon.

**Table S3**. Analysis of variance (p-values) for temperature sensitivity of soil carbon mineralization and soil nitrogen mineralization as influenced by soil depths and treatments.

| Parameter† | Treatment (T) | Temperature (t) | T x t | Depth (D) | T x D | T x t x D |
| --- | --- | --- | --- | --- | --- | --- |
| SCM | <0.001 | 0.009 | 0.004 | 0.004 | 0.004 | 0.03 |
| SCMN | <0.001 | 0.007 | 0.04 | 0.04 | 0.54 | 0.10 |
| SNM | 0.003 | 0.03 | 0.06 | 0.02 | 0.01 | 0.05 |
| SNMN | 0.001 | 0.04 | 0.55 | 0.02 | 0.05 | 0.06 |

†SCM, soil carbon mineralization; SCMN soil carbon mineralization normalized to soil organic carbon; SNM, soil nitrogen mineralization; SNMN soil nitrogen mineralization normalized to total N.
